# Progress in Otology

**Published:** 1903-04-18

**Authors:** 


### PROGRESS IN OTOLOGY.

**Chronic Catarrhal Otitis.**—T. J. Harris, of New York,[1] has published a paper on this subject. He states that no more important addition to our knowledge of this disease has been made in recent years than the demonstration beyond question of its relation to diseases of the nose and naso-pharynx. In the majority of cases the disease can be traced to previous disease of the nose and throat. Two varieties are recognised: (1) chronic hypertrophic catarrh; and (2) chronic hyperplastic catarrh. The hypertrophic form is characterised in general by the presence in the tympanum of secretion, new tissue, and adhesive bands. The recognition of the presence of secretion, which is usually only of brief duration, is an exceptional occurrence. Following the overgrowth of tissue comes the presence of adhesive bands. In diagnosis less importance is attached to the colour of the membrana tympani than was the case formerly. All well-advanced cases will show alterations from the normal light, semi-translucent appearance. The membrane may have a glazed opaque look, or in many instances if the hypertrophic stage is passed there is a thinning or atrophy of its layers. The normal light area will be noticeably increased, or include a large share of the membrane. Chalky deposits may be present. There may be impaired movement of the membrane, or, on the other hand, if atrophy has occurred, certain areas will show excessive movement, particularly the posterior segment. The most characteristic sign is the indrawing of the drum and the prominence of the posterior fold. Slight retraction causes the malleus to be more clearly defined. In some cases from the retraction of the membrane or thinning the incus and inco-stapedial joint can be seen. Tubal catarrh or stenosis is usually present. The normal ear will recognise decidedly lower tones than the catarrhally-affected one, and no point is of greater value in recognising the disease than this. Hyperplastic catarrh differs distinctly from the disease described above. With it there is no preceding attack of aural catarrh and rarely catarrh of the nose or throat. Often the disease affects several members of the same family. The onset is so insidious that the patient cannot usually tell when the disease began. Post-mortem examinations have revealed deposits of bony tissue with anchylosis around the inco-stapedial joint, or affecting the movement of the stapes in the oval window. The otoscope usually shows but little. There may be increased translucency revealing the reddened inner wall of the middle ear. The writer does not speak very hopefully of the results of treatment for either of the forms of disease mentioned, and he suggests no new methods.

**The Operative Treatment of Middle-ear Deafness.** G. Gradinigo[2] has written three articles on this subject. Operative treatment is contra-indicated when the internal ear is also affected, as the reaction following the operation causes an increase of the deafness depending on the inner-ear affection. The cases best adapted for surgical interference are those in which the deafness is due to anchylosis or adhesions of the incus and malleus. The conservative operations, such as simple perforation of the drum or removal of a segment of it, or tenotomy of the tensor tendon, give only a temporary improvement. The author advises removal of the membrana tympani, malleus, incus, and when possible of the stapes. The stapes can only be removed in some cases, and often the foot-plate remains behind after the extraction. Reaction after the operation is less in the catarrhal than in the suppurative cases. After the formation of a new membrane, massage should be used to insure its mobility. The operation as performed by Gradinigo consists of the following steps:—(1) A peripheral circular incision through the membrana tympani carried to within a short distance of the neck of the malleus. (2) Tenotomy of the tensor tympani and division of synechiæ. (3) Incision through the incudo-stapedial joint. (4) Completion of the circular incision to the neck of the malleus and extraction of the latter with the forceps of Faraci. (5) Removal of the incus by means of a forceps or a Zegroni hook. (6) Removal or mobilisation of the stapes, if in sight. With regard to the results of these operations the author's conclusions are as follows. The best results are found in the cases where the diseased condition has resulted from previous suppuration and destruction of the drum membrane. Good results follow where the diseased condition has resulted from suppurative disease without persistent perforation. The treatment had been more successful in the cases of sclerosis than in those of chronic catarrh. The removal of the incus alone is sometimes beneficial.

**Massage of the Ossicula Auditus.**—Milligan[3] has summarised some recent papers on this subject. The method of treatment has been used for certain cases of dry catarrh of the middle ear. Probes like Lucae's pressure probe are used for the purpose, the membrane being previously soaked in cocaine solution. But few patients will tolerate that mode of treatment, owing to the pain it causes. Kœnig recommends dipping the cup-shaped end of a Lucae's probe in paraffin and then applying it to the processus brevi of the malleus. The soft pad of paraffin is said to diminish the pain. Pynchon, discussing the value of pneumatic massage in aural practice (*Laryngoscope*, May 1902), states that slow vibrations, 30 to 90 to the minute, are best adapted for middle-ear troubles. More rapid vibrations, 300 and over, have a greater effect upon the labyrinth. In mixed cases the improvement of the middle-ear condition will favourably affect the labyrinth. Schwabach has found little benefit from vibratory massage in sclerosis of the middle ear, although at times the tinnitus has been improved. Improved hearing and diminished tinnitus was, however, obtained in cases of simple chronic middle-ear catarrh, sub-acute middle-ear catarrh, and post-suppurative otitis media. Breitung's apparatus was used by the author, and the duration of the sittings usually lasted from two to three minutes.

[1] Med. Examiner, October 1902. p. 649. [2] Vide Abst. Amer. Jour. Med. Sci., October 1902, p. 741. [3] Practitioner, November 1902, p. 610.

*(To be continued.)*

